# Transcriptional Regulators and Human-Specific/Primate-Specific Genes in Neocortical Neurogenesis

**DOI:** 10.3390/ijms21134614

**Published:** 2020-06-29

**Authors:** Samir Vaid, Wieland B. Huttner

**Affiliations:** Max Planck Institute of Molecular Cell Biology and Genetics, Pfotenhauerstraße 108, 01307 Dresden, Germany

**Keywords:** neocortex, neurogenesis, transcriptional regulators

## Abstract

During development, starting from a pool of pluripotent stem cells, tissue-specific genetic programs help to shape and develop functional organs. To understand the development of an organ and its disorders, it is important to understand the spatio-temporal dynamics of the gene expression profiles that occur during its development. Modifications in existing genes, the de-novo appearance of new genes, or, occasionally, even the loss of genes, can greatly affect the gene expression profile of any given tissue and contribute to the evolution of organs or of parts of organs. The neocortex is evolutionarily the most recent part of the brain, it is unique to mammals, and is the seat of our higher cognitive abilities. Progenitors that give rise to this tissue undergo sequential waves of differentiation to produce the complete sets of neurons and glial cells that make up a functional neocortex. We will review herein our understanding of the transcriptional regulators that control the neural precursor cells (NPCs) during the generation of the most abundant class of neocortical neurons, the glutametergic neurons. In addition, we will discuss the roles of recently-identified human- and primate-specific genes in promoting neurogenesis, leading to neocortical expansion.

## 1. Introduction

The neocortex is characterized by a six-layered neuronal structure that develops from diverse neural precursor cell (NPC) types. Initially, neuroepithelial (NE) cells, assembled as a pseudostratified epithelium that constitutes the neural plate and then forms the neural tube, undergo symmetric divisions to increase their number [1]. Concomitant with the onset of neurogenesis, NE cells transform into radial glial cells (RGCs), which have the dual function of serving, (i) as the NPCs to all the projection neurons and (ii) as a scaffold for the migration of the newly-generated neurons [2,3,4,5,6,7]. These conversions involve highly-dynamic and tightly-regulated transcriptional regulations, and most of the identified transcriptional programs have been shown to work in concert with one another.

In the first part of this review, we will discuss the current state of knowledge about the transcriptional regulators and their downstream pathways that govern proliferation and differentiation decisions of neocortical NPCs. We will discuss mostly studies that have been done in the mouse model, which is one of the main mammalian model systems for studying neocortical neurogenesis during development. In the second part, we will briefly review the recently-identified human-specific and primate-specific genes whose expression has been shown to be of evolutionary importance in promoting neuron output and expanding the neocortex.

## 2. Neural Progenitor Cell Types in the Developing Neocortex

There are two principal classes of NPCs in the developing neocortex, (i) apical progenitors (APs), whose cell bodies reside in the ventricular zone (VZ) and which undergo mitosis at the ventricular surface and (ii) basal progenitors (BPs), whose cell bodies reside in, and which typically undergo mitosis in, the subventricular zone (SVZ) [7,8,9,10]. BPs have been identified as the primary source of the projection neurons for all layers [11,12,13,14,15] and their abundance is strongly associated with neocortical expansion [16,17,18,19,20,21,22].

After the onset of cortical neurogenesis, APs mainly comprise apical (or ventricular) RGCs (aRGCs), which like their precursor NE cells exhibit apical–basal cell polarity and retain a basal process at mitosis. The BP population is subdivided into two types, (i) basal intermediate progenitors (bIPs), which lack apical–basal cell polarity and cell processes at mitosis and which, at least in the mouse, exhibit limited proliferative capacity [11,23,24,25] and (ii) basal (or outer) RGCs (bRGCs), which retain basal cell polarity, one or more basal- and/or apically-directed cell processes throughout the cell cycle including mitosis, and which exhibit high self-renewal and proliferative capacity [16,17,19,20,26,27,28,29]. bRGCs are abundant in species with an expanded neocortex [16,17,19,26,30,31], whereas in species with a relatively small neocortex such as the mouse, the vast majority of BPs are bIPs and only a minority are bRGCs [20,29,32].

During development, it is important that the relevant types of NPCs are maintained to ensure the proper generation of the various types of neurons. The transcriptional programs that govern neocortical neurogenesis therefore must include the programs that generate and maintain the NPCs committed to neuron production, because any aberration in these programs could cause a reduction in neuron numbers either due to a loss of these NPCs or due to their precocious differentiation. Since the identification of BPs, several studies have sought to identify factors and mechanisms that contribute to their generation, maintenance, and differentiation, with a particular focus on the bRGCs, as these are thought to exhibit a high neuron output capacity [18,21,22,26].

## 3. Transcriptional Programs that Regulate Neocortical Neurogenesis

The earliest known transcriptional programs that are important in triggering the onset of neocortical neurogenesis involve interaction among the bHLH (basic helix–loop helix) family of transcription factors. These very early events of interaction between bHLH transcription factors have been extensively reviewed (please see [33,34,35]). We therefore will only very briefly discuss these bHLH transcription factor interactions. Thus, at the onset of neocortical neurogenesis, live-imaging experiments have shown that the pro-proliferating bHLH genes like *Hes1/Hes5/Hey1* and proneural bHLH genes like *Ngn1/Ngn2* show oscillatory expression in the NPCs [36,37,38]. These bHLH transcription factors compete with each other, and the balance of proliferation vs. differentiation depends on the dynamics of these oscillatory expressions. High Notch signaling leads to the expression of pro-proliferative bHLH transcription factors, which are direct downstream targets of this signaling. These downstream targets promote symmetric NPC proliferation by repressing proneural bHLH transcription factors. A negative autoregulation of transcription and ubiquitin–proteasome-mediated degradation of the protein constitutes an oscillatory degradation mechanism, wherein the phase of decreased expression of pro-proliferative bHLH transcription factors leads to the stable expression of proneural genes, which then promote differentiation of NPCs to become committed to neuron production.

**Figure 1:**
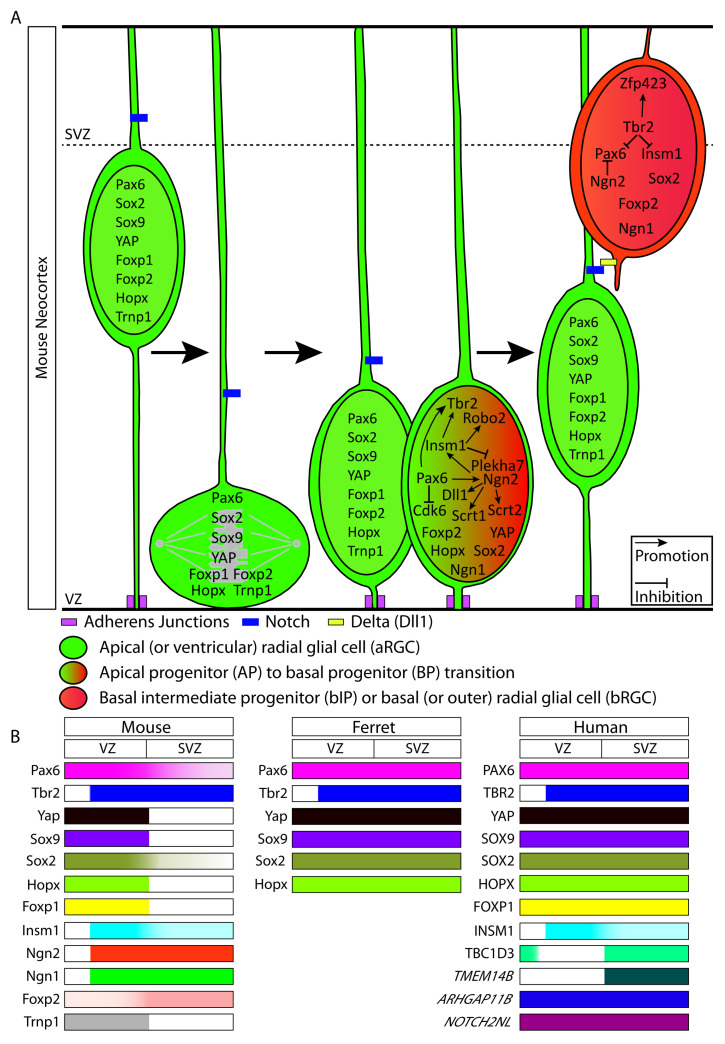
Transcriptional regulators and human-specific/primate-specific genes expressed in neural progenitor cells (NPCs) in the developing neocortex. (**A**) NPC types in the developing mouse neocortex showing the transcriptional regulators expressed in each NPC type. For the NPC types and arrows, see keys. (**B**) Expression pattern of the transcriptional regulators and human-specific/primate-specific genes in the two principal germinal zones, i.e., VZ and SVZ, across mouse, ferret and human. Color intensity in each bar represents the relative expression of the respective protein or mRNA (italics) in the VZ and the SVZ.

We will now discuss the roles of transcription factors and other transcriptional regulators that promote the generation and proliferation of these neuronally-committed NPCs and that influence their capacity for neocortical neurogenesis. Please refer to Figure 1 for an overview of the transcriptional regulators expressed in the various NPC types and in the VZ and SVZ, across mouse, ferret, and human.

### 3.1. Pax6

Pax6 is a member of the Paired Box family of transcription factors that has been shown to regulate brain patterning, aRGC abundance, and symmetric vs. asymmetric NPC division [39,40,41,42,43,44,45,46,47,48].

In addition to its role in aRGC proliferation, Pax6 also positively regulates the expression of neuronal differentiation genes, likely to prepare the NPCs to embark upon a neuronally-committed pathway. Pax6 positively regulates the expression of *Ngn2* by directly binding to an enhancer upstream of the *Ngn2* promoter [49,50,51], and also positively regulates, in a dose-dependent manner, the expression of *Tbr2* [45,52]. At the onset of neurogenesis, NPCs committed to neuron production have been shown to display a significant increase in the length of the G1 phase of their cell cycle [53], and Pax6 has recently been shown to lengthen G1 by decreasing Cdk6 expression [54].

In mouse, Pax6 expression is significantly reduced in BPs compared to aRGCs [55,56,57]. Interestingly, the same Pax6 downstream targets (e.g., *Ngn2* and *Tbr2*) negatively regulate *Pax6* expression to drive neuronal differentiation [13,58,59,60]. However, in contrast to the mouse, Pax6 expression is maintained in the BPs of species with an expanded neocortex [16,17,19,26,31,56,61,62,63,64,65]. How BPs in gyrencephalic species are able to maintain Pax6 expression is not known.

Recently, the functional relevance of maintaining Pax6 expression in BPs has been uncovered. Specifically, using a mouse model, Pax6 expression was specifically sustained in aRGCs that undergo asymmetric division to generate BPs, and in the BPs derived therefrom. Sustained expression of Pax6 increased non-vertical cleavage plane orientation in these BP-genic aRGCs and generated a pool of self-amplifying bRGCs at the expense of bIPs [66]. These bRGCs were shown to exhibit increased cell cycle re-entry, and an increased proportion of them showed either both basal- and apically-directed processes, or an apically-directed process only. Interestingly, these two morphotypes have been reported in the macaque to have a higher self-renewing capacity as compared to the bRGCs that exhibit only a basal process [26]. Additionally, the bRGCs generated upon sustained Pax6 expression were Tbr2− [66]. Taken together, these data underscore the importance of Pax6 expression in generating a bRGC population with primate-like proliferation and self-renewal capacity. Furthermore, upon sustaining Pax6 expression in mouse BPs, the cortical plate was found to be thicker, with an increase in the proportion of Satb2+ upper-layer neurons [66]. Conversely, a Pax6 mouse mutant was shown to exhibit a reduction in the percentage of bRGCs among the BPs [66].

### 3.2. Ngn1 and Ngn2

Ngn1 and Ngn2 are two important class II bHLH transcription factors that are expressed in cortical NPCs. Loss-of-function and gain-of-function studies have revealed a proneural function for both of these genes [51,58,60,67,68,69,70]. During early neocortical neurogenesis in the mouse, both Ngn1 and Ngn2 show strong expression throughout the VZ in the dorsal telencephalon [51,69,71]. However, by E15.5, the expression level of Ngn1 goes down, whereas Ngn2 continues to be highly expressed [51,69,71].

Ngn1 has recently been shown to regulate the differentiation of NPCs at the early stage of cortical neurogenesis. During early cortical neurogenesis in the mouse (E12.5–E13.5), *Ngn1*-mutant aRGCs produce more Tbr2+ bIPs which show an increased rate of production of deep-layer neurons as identified by Tbr1 and Ctip2 [71]. Consistent with the absence of Ngn1 expression during late neurogenesis, the bIPs after E15.5 were similar between wildtype and *Ngn1*-mutant mouse embryos, demonstrating that Ngn1 is not required for the late stage of neocortical neurogenesis [71]. Interestingly, at E15.5, the number of deep-layer neurons became similar between wildtype and *Ngn1*-mutant mouse embryos [58,71], suggesting that Ngn1 functions to maintain the pace of neocortical neurogenesis during early developmental stages.

Ngn2 has been shown to be both necessary and sufficient to specify a glutamatergic neuronal identity [51,69,70]. In contrast to Ngn1, Ngn2 expression mediates transition of aRGC to the SVZ [24,72], resulting in the generation of basally-dividing Tbr2+ bIPs [24,72]. Ngn2 (i) promotes the expression of insulinoma-associated 1 (Insm1), a zinc-finger transcriptional regulator of the SNAG family that has been implicated in the generation of BPs [73,74]; (ii) promotes the expression of Scratch 1 and Scratch 2, two other members of the SNAG family that are implicated in triggering the onset of migration of BPs and newly-generated neurons [75]; (iii) directly activates the expression of Tbr2 [51,60,72,76]; and (iv) represses Pax6 expression [58,60,72].

Despite its continual expression throughout the neurogenic period, loss-of-function studies in the mouse have demonstrated that, similar to Ngn1, Ngn2 is required to specify the identities of deep-layer neurons but not upper-layer neurons [51,69]. The Ngn2-mediated transition of aRGCs to Tbr2+ bIPs has been observed only for early stages of neocortical neurogenesis, i.e., until E13.5 [72]. After E14.5, the Ngn2 protein is still expressed but is phosphorylated by glycogensynthase kinase 3β. This phosphorylation promotes the heterodimerization of Ngn2 with Tcfe2a, a class I bHLH transcription factors, and this heterodimer exhibits a strongly-reduced Ngn2 transcriptional activity. Due to this reduction, overexpression of Ngn2 from E14.5 onwards does not cause any additional transition of aRGCs to the SVZ [72].

Ngn2 promotes bIP generation only during early neocortical neurogenesis. However, the fact that in mouse embryos Tbr2+ bIPs are also produced after E14.5 suggests that the mechanisms regulating the generation of bIPs, and of BPs in general, likely involve other genes with similar function. It is possible that bIP generation is regulated in a sequential manner by different developmental stage-specific gene expression programs. Similar to the role of Ngn2 in early neurogenesis, it is possible that the genes with essential roles in late neurogenesis may participate in late bIP generation, either by promoting similar downstream mechanisms of BP generation as those operating in early neurogenesis, or by being part of other machineries.

### 3.3. Insulinoma-Associated 1 (Insm1)

Insm1 is a zinc-finger transcriptional regulator belonging to the SNAG family of proteins that has been implicated in the generation of BPs [73,74,77]. The importance of this gene in promoting neurogenesis is underscored by the fact that its expression is turned on specifically in BP-genic aRGCs and newly-generated BPs, remains high during the entire period of neocortical neurogenesis, and is turned off in the newborn neurons [73]. In proliferating aRGCs, which have not yet switched to BP generation, *Insm1* expression is likely blocked via Hes5 [73]. Consistent with its role specifically in neurogenic NPCs, *Insm1* expression is positively regulated by Ngn2. However, additional genes are likely to also contribute to the induction of Insm1 expression because *Insm1* expression is not completely abolished in *Ngn2*-null-mutant mice [73]. Retinoic acid signaling seems to be another specific regulator of *Insm1* expression [78], but whether retinoic acid signaling works redundantly or synergistically with Ngn2 is not known.

Insm1 protein expression was found to be higher in the VZ than SVZ, with most of the Insm1+ cells in the VZ being newly-generated BPs [74]. Knock-out and overexpression studies have shown that Insm1 is both necessary and sufficient for BP generation [73,74]. However, a significant number of BPs are still produced in *Insm1*-null-mutant mice, suggesting that although Insm1 promotes BP generation, its role is not absolutely essential for this purpose. An important finding regarding the role of Insm1 in promoting neurogenesis was that upon Isnm1 overexpression in embryonic mouse neocortex, about 40% of the BPs were bRGCs [73,74]. This is consistent with a possible role of Insm1 in the expansion of the neocortex, as bRGCs are the principal neuron-producing NPC type in species with an expanded neocortex [16,17,19,26,30,31,65].

In terms of the mechanism underlying the increase in BP generation, Insm1 was shown to down-regulate the expression of Plekha7, an apical adherens junction belt-specific protein, causing the aRGCs to delaminate and become bRGCs [74]. In addition to repressing Plekha7, Insm1 was shown (i) to promote the expression of Robo2 [73], a transmembrane receptor of the ROBO family that is implicated in positively regulating production and detachment of BPs from the apical side [79,80] and (ii) to promote the expression of Tbr2 [73], thus triggering the first steps of aRGC to BP transition.

### 3.4. Tbr2

Tbr2 is a member of the T-box gene family and promotes the generation, maintenance, and differentiation of bIPs [55,59,81,82,83,84,85]. Several transcriptional regulators have been identified that positively regulate Tbr2 expression in bIPs [45,60,72,73,76,86,87]. Tbr2 plays an essential role in the amplification of bIPs and thereby in expanding the neuron output derived from these NPCs [59]. The Tbr2 protein is expressed from very early G1 in the newly-formed bIPs migrating through the VZ [88], and continues to be expressed in the bIPs residing in the SVZ [55,84,85], but is largely absent in the more proliferative bRGCs of certain species such as human. Tbr2 overexpression in developing ferret cortex has been shown to induce additional folds and fissures, emphasizing its role in promoting neurogenesis and in the expansion of the neocortex [89]. Deletion of Tbr2 in mouse does not result in the complete loss of bIPs, but their neurogenic efficiency is significantly reduced [13]. Consistent with this, recent independent analyses of the Tbr2+ lineage have shown that during mouse cortical development, the vast majority of glutamatergic neurons, across the various cortical layers, transit through a Tbr2+ intermediate state [13,14,15].

A recent single-cell transcriptome study has shown heterogeneity in gene expression patterns among the BP population, with at least two subpopulations existing at E14.5 in embryonic mouse neocortex [88]. Further in-situ hybridizations showed that the Tbr2+ subpopulation observed in the VZ (likely the newly-generated BPs) expressed genes like *Afap1/Hes6* suggesting a less-differentiated state. In contrast, the Tbr2+ BP subpopulation residing in the SVZ showed expression of neuronal differentiation markers, such as Neurod1/Nrn1/Mgat5b [88]. The findings that the Tbr2 protein is expressed very early in the cell cycle and is present in both less-differentiated and more-differentiated BP subpopulations [88] raise the possibility that certain Tbr2 downstream targets may also follow this pattern. This in turn would be consistent with the concept that, at least in mouse, Tbr2 promotes neurogenesis by mediating the transition from aRGCs to bIPs, with a gradual shut-down of the aRGC program and the turning on of a neuronal differentiation program.

In addition, Tbr2 represses Zfp423, a cofactor necessary for neuronal differentiation in response to retinoic acid signaling, to prevent premature neuronal differentiation [90]. Furthermore, Tbr2 down-regulates *Pax6* and *Insm1* expression [13,91]. Taken together, these reports elegantly illustrate how a single transcription factor fine-tunes gene expression to allow a smooth fate transition among NPC types. Finally, Tbr2 seems to keep the total number of neurons generated under control as about 33% of Tbr2 lineage-derived cells die [15] via apoptosis or phagocytosis [92].

### 3.5. Foxp1 and Foxp2

The forkhead box P (Foxp) protein subfamily belongs to the Fox family of transcription factors, with Foxp1, Foxp2, and Foxp4 being highly expressed in the central nervous system and known to regulate brain development and function [93,94,95,96,97,98]. Although the role of the Foxp subfamily in neuron migration, maturation, and circuit formation has been extensively studied, its role during embryonic cortical neurogenesis was uncovered only very recently, albeit with conflicting results.

Braccioli et al. showed that Foxp1 is required for neuronal differentiation [98]. Upon a shRNA-mediated *Foxp1* knock-down in embryonic mouse neocortex, the number of Tbr2+ bIPs was increased but the number of Ctip2+ neurons was decreased [98]. Consistent with a role in positively regulating neuronal differentiation, Foxp1 was found to directly bind to the *Jag1* promoter and to repress its expression. In the *Foxp1* knock-down, the Notch ligand Jag1, the Notch intracellular domain (NICD), and the downstream effector Hes1 showed increased expression [98].

Contrary to this, using a similar shRNA-mediated *Foxp1* knock-down, Li et al. showed that neuronal differentiation is unaffected [99]. It is important to note that in both these reports a consistent and similar phenotype of neuronal migration was observed. Since shRNA-mediated knock-downs are prone to off-target phenotypes, Li et al. used the more specific siRNAs for additional knock-downs, but the results obtained with these additional knock-downs were compared only with regard to the migration defects and not the neuronal differentiation defects [99].

Pearson et al. showed that expression of *Foxp1* in embryonic mouse neocortex goes down by about 3.5-fold after E12.5. A *Foxp1* knock-out during early cortical neurogenesis (until E13.5) decreases Pax6+ cells and increases Tbr2+ cells [100]. Consistent with the low expression of Foxp1 after E13.5, a conditional knock-out of *Foxp1* does not have any effect on the generation of Pax6+ or Tbr2+ cells at late stages of cortical neurogenesis (after E14.5) [100], a result consistent with the findings of Li et al. [99].

Similar to *Foxp1*, *Foxp2* manipulations have been performed in embryonic mouse neocortex to understand the role of this gene in cortical neurogenesis. Foxp2 is expressed in both Pax6+ aRGCs in the VZ and Tbr2+ bIPs in the SVZ, with the Tbr2+ cells in the SVZ showing heterogeneity, i.e., high and low levels, of Foxp2 expression [94]. shRNA-mediated knock-down of *Foxp2* in embryonic mouse neocortex at E13.5 delayed the transition of aRGCs to bIPs and impaired the migration of neurons to the upper neocortical layers [94]. The aRGC-to-bIP transition delay did not cause any long-term consequences for the number of neurons generated because an analysis at the postnatal stage (P3) showed that eventually normal numbers of neurons were generated upon *Foxp2* knock-down. Interestingly, although the human and murine FOXP2 proteins are highly homologous to each other, with only three amino acids being different between the two species [101], overexpression of human, but not mouse, FOXP2 increased the transition rate of aRGCs to bIPs [94]. This demonstrates a functional difference between the human and murine FOXP2 protein.

The human FOXP2 protein has been implicated in the acquisition of language by humans, a topic not further discussed here as it has been extensively covered elsewhere [102,103].

### 3.6. Yes-Associated Protein (YAP)

Several reports have established a role for the yes-associated protein 1 (Yap1 or YAP), a transcriptional regulator controlled via phosphorylation and the major down-stream effector of the Hippo pathway, in aRGC proliferation [104,105,106,107,108]. A recent report has extended these studies to BPs, examining a possible role of YAP in the maintenance and proliferation of these NPCs. Specifically, it was shown that the BPs in the developing neocortex of gyrencephalic species like ferret and human show high expression of nuclear, non-phosphorylated (active) YAP, whereas this was not the case for embryonic mouse neocortex [109]. Disruption of *YAP* expression in fetal human neocortex and inhibition of YAP function in embryonic ferret neocortex reduced BP abundance. Conversely, conditional expression of a constitutively-active YAP in the BP lineage of embryonic mouse neocortex increased the proliferative capacity of BPs and resulted in increased upper-layer neuron generation [109]. Similar to sustained Pax6 expression, but in contrast to Insm1 overexpression, the increased level of BPs observed upon conditional expression of a constitutively-active YAP in the BP lineage showed a decreased proportion of Tbr2+ and an increased proportion of Sox2+ BPs. A similar result of increased proliferation in the SVZ was reported in a very recent, independent study in which *Yap* mRNA (mYAP) instead of constitutively-active YAP was overexpressed in the NPCs of embryonic mouse neocortex [110]. However, in contrast to Kostic et al. [109], Mukhtar et al. [110] did not report a decrease in Tbr2+ cells in the SVZ when mYAP was overexpressed.

### 3.7. Sox2 and Sox9

Sox2 and Sox9 are members of the Sox (Sry HMG-box) family of transcription factors [111]. Sox2 has been extensively studied for its role in NPC proliferation, and we will not go into further details here (please see [112], for an excellent comprehensive review).

Sox9 has been shown to be essential for the regulation of both neuron and glia differentiation in the developing brain [113,114,115,116,117,118,119,120], and a recent report [121] has extended its role to BP proliferation. Sox9 is highly expressed in the VZ of developing mouse, ferret, and human neocortex and in the SVZ of developing ferret and human neocortex but is not expressed in the mouse SVZ [121,122]. Contrary to the embryonic mouse neocortical SVZ, Sox9 is highly expressed in the BPs residing in the inner subventricular zone (ISVZ) and outer subventricular zone (OSVZ) of ferret and human developing neocortex, with almost all bRGCs positive for Sox9 [121]. These Sox9+ BPs are highly proliferative and are capable of cell-cycle re-entry. *Sox9* knock-out in embryonic ferret neocortex and conditional expression in embryonic mouse neocortex demonstrated that Sox9 is both necessary and sufficient for BP proliferation.

Interestingly, conditional Sox9 expression in mouse BPs increased the proliferation and cell cycle re-entry of these NPCs in both a cell-autonomous and cell-non-autonomous manner. Whereas the cell-autonomous expression drove the proliferating BPs towards gliogenesis, the cell-non-autonomously proliferating BPs continued neurogenesis, eventually generating more upper-layer neurons [121]. This dual role of Sox9 could be particularly important and beneficial in species like human, where both neurogenesis and gliogenesis occur to a large extent, simultaneously. Sox9 was found to increase the expression of ECM-related genes [121], notably of laminins, which likely explains the cell-autonomous and cell-non-autonomous increase in BP proliferation, as ECM components have previously been implicated in promoting NPC proliferation [16,57,123,124,125,126,127,128].

### 3.8. Hopx

The homeodomain-only protein (HOPX) is the smallest known member of the homeodomain-containing protein family [129,130,131,132], but unlike other homeodomain-containing transcription factors, it lacks the ability to bind DNA. Hopx gained interest regarding a possible role in neocortical development when it was identified as a bRGC marker in the developing human neocortex [133,134]. A recent report has then uncovered a role of Hopx in promoting neurogenesis in developing neocortex [32]. Thus, similar to HOPX expression in fetal human neocortex, Hopx was found to be expressed in NPCs of developing ferret and mouse neocortex. Using embryonic mouse neocortex as a model, it was further shown that Hopx does not affect the proliferation of aRGCs but is both necessary and sufficient to increase the relative abundance of bRGCs among the BPs, and therefore is a key determinant for bRGC expansion [32]. In line with this, overexpression of Hopx in the postnatal mouse SVZ has been shown to induce bRGC generation [135]. Consistent with increasing BP proliferation, overexpression of Hopx increased the generation of Satb2+ upper-layer neurons, whereas *Hopx* knock-down decreased the generation of upper-layer neurons [32].

An important insight into the evolution of the neocortex was obtained when the expression of Hopx was compared between the embryonic mouse lateral and medial neocortex [32]. Specifically, the bRGCs in the mouse medial neocortex were not only found to be more abundant than in mouse lateral neocortex, but also to exhibit a gene expression profile more similar to human bRGCs than those in lateral neocortex [32]. Since Hopx was found to be required to maintain the bRGC levels in the mouse medial neocortex, these data suggest a key role of Hopx in generating primate-like bRGCs. On a more general note, given that the mouse neocortex is thought to be secondarily lissencephalic, i.e., to have evolved by the secondary loss of gyrification [32], the data by Vaid et al. imply that the mouse medial neocortex may be more closely related to an ancestral gyrencephalic neocortex than the mouse lateral neocortex.

### 3.9. Trnp1

Trnp1 encodes a nuclear DNA-binding protein [136]. Stahl et al. showed that before the onset of cortical neurogenesis in mouse, Trnp1 is expressed in all NE cells, but after the onset of cortical neurogenesis Trnp1 is restricted to a subset of Pax6+ aRGCs and is absent in BPs [137]. They further showed that in-vivo Trnp1 overexpression increased Pax6+ aRGCs and reduced the generation of Tbr2+ bIPs. Conversely, shRNA-mediated *Trnp1* knock-down led to the delamination of Pax6+ aRGCs and increased the generation of both bIPs and bRGCs, with a greater proportion of bRGCs than bIPs among the BPs [137]. Similar to sustained Pax6 expression, *Trnp1* knock-down increased non-vertical cleavage plane orientations of aRGCs to cause these NPCs to delaminate and generate increased levels of bRGCs which were Tbr2−. However, unlike in the case of sustained Pax6 expression, *Trnp1* knock-down induced neocortical folding of embryonic mouse neocortex [137].

In the human, *TRNP1* showed high expression in the VZ and a relatively lower expression in the SVZ [137]. In the ferret, *Trnp1* showed a dynamic expression, as follows. At E34, *Trnp1* showed a high expression in the VZ and very low to no expression in the SVZ, whereas at P1, *Trnp1* expression in the VZ was higher than at E34 and similar between the VZ and SVZ [138]. These authors further showed that during ferret neocortical development, in-vivo Trnp1 overexpression reduced the abundance of bRGCs, with a concomitant increase in aRGC abundance. Conversely, expression of a dominant-negative Trnp1 increased the abundance of bRGCs and reduced the abundance of aRGCs. Martinez-Martinez et al. also showed that a down-regulation of Cdh1, which encodes a type-1 cadherin, is essential to allow bRGC production in the ferret, with simultaneous down-regulation of *Trnp1* being essential for Cdh1-mediated generation of bRGCs [138].

## 4. Human–Specific and Primate–Specific Genes that Promote Neocortical Neurogenesis

The above-mentioned examples highlight the transcriptional regulators that, notably in the mouse embryo, promote neocortical neurogenesis by increasing the production of NPCs, with some examples even pertaining to NPCs that are typically found in species with an expanded neocortex, like primates. The latter is important in light of the fact that such species typically generate many more cortical neurons than the mouse. In addition to the transcriptional regulators themselves that are encoded by a given genome and the focus of the present review, other ways to promote neurogenic output during cortical development are (i) by enhancing the expression of conserved pathways by novel enhancers, (ii) by introducing new genes over the course of evolution that can either work synergistically with existing genes or can work in their own capacity to enhance neurogenesis, or (iii) by changing the epigenetic state.

Recently, several transcriptomic studies have aimed at identifying transcriptional changes that underlie the evolutionary expansion of the neocortex in primates and more specifically in the human lineage [124,125,133,134,137,139,140]. Among the various human-specific and primate-specific genes identified, only a few have so far been tested for their possible role in promoting cortical neurogenesis, as is discussed below. Please refer to Figure 1B for an overview of the human-specific and primate-specific genes expressed in the human VZ and SVZ.

### 4.1. ARHGAP11B

*ARHGAP11B* is the first identified human-specific gene shown to underlie BP expansion [125]. ARHGAP11B originated ≈ 5 mya by partial duplication of an ancestral gene, ARHGAP11A [141,142]. Subsequently, a point mutation introduced a new splice donor site that gave rise to a novel, human-specific C-terminal protein sequence in ARHGAP11B [143]. Overexpression of *ARHGAP11B* in embryonic mouse and ferret neocortex was shown to expand the pool size of BPs [125,143,144]. Interestingly, *ARHGAP11B* overexpression generated Tbr2+ BPs in the mouse model [125,143], but generated more primate-like Tbr2− bRGCs in the ferret model [144]. In developing ferret neocortex, *ARHGAP11B* overexpression in addition extended the length of the neurogenic period and consequently the proportion of Satb2+ neurons in the upper layers [144]. Very recently, it was shown that ARHGAP11B is essential for BP proliferation in fetal human neocortex, localizes to mitochondria, and induces a metabolic shift to glutaminolysis to expand the BP pool [145]. *ARHGAP11B* therefore is a paradigmatic example highlighting that among the transcriptional changes in primate evolution that promote neocortical neurogenesis, a gene related to metabolism plays a major role.

### 4.2. NOTCH2NL

Three independent reports identified NOTCH2NL as another human-specific gene that promotes NPC, and notably BP, proliferation [146,147,148]. NOTCH2NL is a paralog of NOTCH2 and is expressed in the bRGCs residing in the SVZ/OSVZ of the developing human neocortex [146,147,148]. In-vitro expression of *NOTCH2NL* in human NPCs, at a time when it is not expressed, led to their clonal expansion and increased neuronal output [147]. Conversely, *NOTCH2NL* deletion in human cerebral organoids reduced their size and caused premature differentiation [148]. Consistent with this, overexpression of *NOTCH2NL* delayed the differentiation in mouse cerebral organoids [148]. In-vivo expression of *NOTCH2NL* in developing mouse neocortex expanded Pax6+ aRGCs in the VZ [147] and Tbr2+ bIPs in the SVZ [146]. It was further shown that NOTCH2NL interacts with the Notch receptor NOTCH2 and with the Notch ligand DLL1 to increase Notch signaling that promotes proliferation of cortical NPCs [147,148].

### 4.3. TBC1D3

*TBC1D3* is a hominin-specific gene, encoding a protein of the RABGAP family, which has multiple copies present in the human genome, a single copy in the chimpanzee genome, and is absent in other primates and mammals [149,150,151]. *TBC1D3* paralogs have been shown to be expressed in the human brain [149,152], with high TBC1D3 expression in the aRGCs very near to the ventricular surface and in the BPs residing in the OSVZ [153]. Interestingly, TBC1D3 expression in embryonic mouse neocortex caused significant reduction in the expression of Trnp1 and of Cdh2, a type-1 cadherin, and caused delamination of aRGCs to generate bRGCs [153]. Hence, the generation of bRGCs is achieved by a simultaneous down-regulation by TBC1D3 of two inhibitors of bRGC generation. The delaminated aRGCs overwhelmingly generated proliferating bRGCs (about 40%) [153] and represented all the various bRGC morphotypes that have been reported in developing primate neocortex [26]. These phenotypes of TBC1D3 expression are very similar to the sustained Pax6 expression phenotype [66]. TBC1D3 was shown to increase ERK signaling in the bRGCs, which is likely to confer them a high proliferative capacity [153]. Interestingly, ERK signaling-mediated phosphorylation of Pax6 has been shown to increase transcriptional activity of Pax6 [154]. Consistent with the increased abundance of NPCs, *TBC1D3*-transgenic mice showed enhanced cortical neurogenesis with a specific increase in the number of upper-layer neurons [153].

### 4.4. TMEM14B

Liu et al., by RNA sequencing on fetal human brain samples, identified *TMEM14B*, along with other genes like *KCNK10*, *DAG1*, and *HP1BP3*, as a primate-specific and bRGC-specific gene [140]. *TMEM14B* expression in embryonic mouse neocortex (i) expanded the SVZ, (ii) increased Pax6+ Hopx+ and Sox2+ Hopx+ primate-like bRGCs, and (iii) increased Tbr2+ bIPs. This expression increased cell cycle re-entry of the BPs by reducing the cell cycle length. *TMEM14B* overexpression increased the overall cortical thickness, with an increase in both deep-layer and upper-layer neurons. TMEM14B was shown to interact with the Ras-activating-like protein IQGAP1, with IQGAP1 expression in embryonic mouse neocortex partially phenocopying the *TMEM14B* overexpression phenotype. TMEM14B was further shown to promote the phosphorylation and subsequent nuclear translocation of IQGAP1 [140].

## 5. Concluding Remarks

This review offers a comprehensive view of our current understanding of transcriptional regulators that govern neurogenesis by regulating the generation, maintenance, amplification, and differentiation of NPCs. First, the transcription factors Pax6 and Tbr2 are paradigmatic examples of transcriptional regulators operating in the AP-to-BP lineage. Second, the generation of self-amplifying bRGCs with primate-like morphology and behavior upon expression of certain transcriptional regulators in the mouse model suggests that the underlying genetic toolkit that can increase neurogenesis, a feature common in species with an expanded neocortex, is actually very conserved. Third, the identification of human-specific and primate-specific genes that are able to promote neurogenesis when introduced into the mouse model provides evidence for the concept that the neurogenesis-promoting evolutionary changes utilize pre-existing mechanisms and do not necessarily need the introduction of novel downstream processes.

Our knowledge about the interplay between the transcriptional regulators that govern the generation of bRGCs is still limited. For example, on the one hand, studies on Pax6 and Insm1 have provided insight into how bRGCs with high proliferative capacity can be generated, but on the other hand, have raised new issues as the bRGCs generated by Pax6 or Insm1 overexpression differ in the downstream programs that get activated (Tbr2− lineage in Pax6, but Tbr2+ lineage in Insm1) [66,73,74]. Hence, the transcriptional programs that drive the generation of highly proliferative vs. neuronally-committed bRGCs appear to be more diverse than previously thought. These examples therefore call for a more comprehensive analysis of the phenotypes and of the lineages of the NPCs generated upon manipulation of transcriptional programs. Identification of more bRGC-generating genes will further refine our understanding of how different transcriptional programs interact with respect to their potential to confer high neuron output capacity to the NPCs.

Finally, the example of Ngn1 and Ngn2 demonstrates that the progression from early to late neurogenesis is not simply a temporal extension of the early neurogenesis program, but likely involves further qualitative changes in the transcriptional program. This is of interest given the fact that the generation of upper-layer neurons is not only a hallmark of late neurogenesis, but of the evolutionary expansion of the neocortex.

## Abbreviations

APs	Apical progenitors
aRGCs	Apical (or ventricular) RGCs
bRGCs	Basal (or outer) RGCs
bIPs	Basal intermediate progenitors
BPs	Basal progenitors
bHLH	Basic helix-loop-helix
Foxp	Forkhead box p
Hopx	Homeodomain-only protein
ISVZ	Inner subventricular zone
Insm1	Insulinoma-associated 1
NPCs	Neural precursor cells
NE	Neuroepithelial
NICD	Notch intracellular domain
OSVZ	Outer subventricular zone
RGCs	Radial glial cells
SVZ	Subventricular zone
VZ	Ventricular zone
YAP	Yes-associated protein
